# Editorial: Learning Analytics – Trends and Challenges

**DOI:** 10.3389/frai.2022.856807

**Published:** 2022-02-25

**Authors:** Aleksandra Klašnja-Milićević, Mirjana Ivanović, Boban Vesin, Maya Satratzemi, Barbara Wasson

**Affiliations:** ^1^Department of Mathematics and Informatics, University of Novi Sad, Novi Sad, Serbia; ^2^Department of Business, History and Social Sciences, University of South-Eastern Norway, Notodden, Norway; ^3^Department of Computer Science, Norwegian University of Science and Technology, Trondheim, Norway; ^4^Department of Applied Informatics, University of Macedonia, Thessaloniki, Greece; ^5^Department of Information Science and Media Studies, University of Bergen, Bergen, Norway

**Keywords:** learning analytics, artificial intelligence, data mining, learning strategies, teaching strategies

Learning analytics aims to collect and analyse data from students and learning environments to support learning on different levels. Although learning analytics is a relatively new area, it has matured significantly, particularly in its application in higher education. This Research Topic is devoted to the issues of collecting, analyzing, evaluating, reporting, and utilizing data to improve learning. The emphasis is on cases that are new to study, unique in the application, or significantly increased in scope. The Topics' authors emphasized the need for more research and work not only on automatically gathering enormous amounts of data from learners but also in giving meaningful insights, sense-making, and applying it for further improvements in learning processes. Students, instructors, and administrators may improve learning and course results by harnessing learning analytics, for example, by generating more engaging and effective teaching and learning strategies. Learning analytics not only provides feedback to individual students and instructors but may also reveal trends across schools or within specific programs subject disciplines, across class sizes, and in other settings. Each audience's analytics questions, insights, and perspectives help educate and drive the design and delivery of integrated analytics to each of these analytics audiences.

The first article, “*Applications of learning analytics in high schools: A Systematic Literature Review*,” by de Sousa et al., reports the results of a literature review focused on the adoption of learning analytics in high schools and presents the main approaches, techniques and challenges of adopting learning analytics in that context. As the main educational goals in using LA in high schools, the authors identified: predicting and enhancing student learning outcomes; analyzing students' learning processes; reinforcing teacher's decisions and reflection; and supporting writing activities. In addition, the distillation of data for human judgment, prediction, relationship mining, discovery with models, and clustering were identified as the main approaches for the use of LA. It was found that the majority of the papers presented few or no details that could ensure some level of confirmation on the positive impact of LA in a high school context. To broaden LA applications in high schools, the authors proposed several research directions mainly concerned with used methodological processes and mining techniques.

The second article, “*Sentiment Analysis of Students' Feedback in MOOCs*” by Dalipi et al., reflects the challenges of collecting, analyzing, reporting and utilizing data with the specific intent to improve learning. Four parts included in this Research Topic indicate the significant potential of learning analytics for improving learning and teaching. Dalipi et al., in their literature review, explored the use of sentiment analysis for evaluating students' feedback in MOOCs. Sentiment analysis techniques have been identified as an essential factor in understanding students' feedback in MOOCs, enhancing the learning experience, and improving teaching by analyzing the learners' behavior toward courses, platforms, and instructors. The authors identified significant research themes and provided recommendations and directions for future research.

The third article*, “Keep Calm and Don't Carry-Forward: Toward sensor-data driven AI agent to enhance human learning”* by Sharma et al., presented results of the extensive experiments conducted on an *in-situ* study where 40 children, age 9–12, played Motion Based Educational Games for math and language development. The research was motivated by the complexity of the real-time use of student-generated Multimodal Data derived from their interactions with embodied learning systems and how to handle the sensor data and enable an AI agent to use them. During gameplay, the students' experiences were unobtrusively and continuously monitored using eye-tracking glasses, physiological wristbands, and Kinect. In such a way, the different cognitive and physiological dimensions of students' progress were understood *via* the “see-solve-move-respond” cycle. The authors proposed the innovative Carry Forward Effect whereby students propagate the cognitive and physiological effects derived from their Multimodal Data. Presented results validate the importance of wristband and eye-tracking data as key indicators for prioritizing adaptive feedback to support students in Motion-Based Educational Games.

The fourth article, “*Explainable AI for Data-Driven Feedback and Intelligent Action Recommendations to Support Students Self-Regulation*,” by Afzaal et al., presents research that combines a prediction approach with an explainable machine learning (ML) approach. In particular, they apply explainable ML to computer automatic and intelligent feedback provision that provides information feedback and actionable recommendations for students. Using data from a Learning Management System (LMS), the researchers developed an explainable ML algorithm that builds a predictive model that first predicts students' performance in each assignment and quiz. Second, it uses the model to determine feedback on assignments or quizzes. The ambition is to provide automatic data-driven feedback in the form of recommendations that may help students self-regulate, adjust, and target their engagement in the course, thus adjusting their learning effects. To examine the effectiveness of the approach, a dashboard was designed, developed and implemented in a real course setting. Evaluation of the dashboard included perceptions of its utility and benefits. The results showed that the dashboard helped students in self-regulation, boosted their motivation, and improved their academic performance.

## Author Contributions

All authors listed have made a substantial, direct, and intellectual contribution to the work and approved it for publication.

## Conflict of Interest

The authors declare that the research was conducted in the absence of any commercial or financial relationships that could be construed as a potential conflict of interest.

## Publisher's Note

All claims expressed in this article are solely those of the authors and do not necessarily represent those of their affiliated organizations, or those of the publisher, the editors and the reviewers. Any product that may be evaluated in this article, or claim that may be made by its manufacturer, is not guaranteed or endorsed by the publisher.

